# Akt在非小细胞肺癌中作用的研究现状

**DOI:** 10.3779/j.issn.1009-3419.2010.11.12

**Published:** 2010-11-20

**Affiliations:** 610041 成都，四川大学华西医院呼吸内科 Department of Respiratory Medicine, West China Hospital of Sichuan University, Chengdu 610041, China

**Keywords:** Akt, PDK1, Raf-1, 肺肿瘤, Akt, PDK1, Raf-1, Lung neoplasms

## Abstract

肺癌是目前世界上最常见的恶性肿瘤之一，但其发病机制尚不完全清楚。Akt是一种重要的信号通路关键蛋白，广泛参与肿瘤细胞的生长、增殖、凋亡及侵袭等过程。本文就Akt及其重要的上下游调节分子之——PDK1、Raf-1和p70S6K在非小细胞肺癌中的作用研究现状做一综述，以期为阐明非小细胞肺癌的发病机制提供新的依据。

## Abstract (en)

Lung cancer is one of the most common malignant tumors in the world, but its pathogenesis has still been remaining confusing. As an important protein in several signaling pathways, Akt has been identified to play a major role in the growth, proliferation, apoptosis and invasion of tumor cells. This paper is to review the effects of Akt, together with PDK1, Raf-1 and p70S6K, which are upstream and downstream regulatory molecules of Akt, and provide a new basis for the pathogenesis of non-small cell lung cancer.

肺癌是目前对人类健康威胁最大的恶性肿瘤之一，全球每年约有135万人被确诊为肺癌，120万人死于肺癌^[1]^。虽然医学技术有了长足发展，但肺癌患者的5年生存率并未得到明显改善，仅为15%左右^[1]^。其根本原因在于肺癌的发病机制尚不清楚、临床缺乏有效的早期诊断和治疗手段。病理学上，肺癌分为小细胞肺癌（small cell lung cancer, SCLC）和非小细胞肺癌（non-small cell lung cancer, NSCLC），后者约占80%-85%。20世纪90年代以来，信号传导通路逐渐成为肿瘤学研究领域的热点。丝氨酸/苏氨酸蛋白激酶B（protein kinase B, PKB/Akt）因其处于多条信号通路的交叉点而受到广泛关注。

## 1 Akt的研究现状

Akt是存在于人类染色体基因组中鼠类胸腺淋巴瘤病毒（T-8 strain from AKR/J mouse, AKT8）致癌基因（v-Akt）的同源物，其编码的蛋白质Akt是一种丝氨酸/苏氨酸蛋白激酶，因与蛋白激酶A、C高度同源，又名蛋白激酶B（protein kinase B, PKB）^[2]^。Akt经磷脂酰肌醇3-激酶（phosphatidylinositol 3-kinase, PI3K）活化后，磷酸化其下游多种底物，参与细胞生物学功能的调节，影响细胞生物学行为，主要表现为促进细胞增殖、抑制细胞凋亡、提高细胞的乏氧耐受性、促进肿瘤细胞侵袭转移和组织血管生成等，从而参与多种肿瘤的发生^[3]^。随着研究的深入，现已基本绘制了以Akt为中心的信号通路图（图 1）。

**1:**
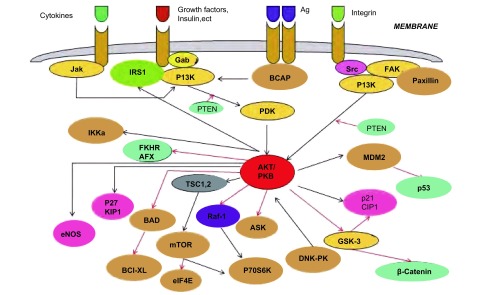
Akt信号通路图 Signaling pathway of Akt

Akt家族包括Akt1、Akt2、Akt3三种亚型，其中Akt2是最重要的一种，不仅具有Akt的普遍特征，还有其独特的生物学功能，主要介导肿瘤细胞的粘附、运动、侵袭和转移^[4-6]^。有学者研究发现，约10%-20%的原代胰腺癌细胞和胰腺癌细胞株存在Akt2的扩增或过表达。当用Akt2反义核苷酸转染胰腺癌细胞后，肿瘤生长明显受抑，且仅局限于管腔内；而导入正义核苷酸的对照组肿瘤细胞，其生长则无明显变化，并侵入管壁。此现象表明Akt2的过度表达促进了胰腺癌细胞的生长和侵袭^[7-10]^。Rychahou等^[11]^用qRT-PCR的方法检测了86例结肠癌患者肿瘤组织和正常结肠组织中Akt2 mRNA的水平，发现肿瘤组织中的Akt2 mRNA是正常组织的8倍-10倍；随后对36例有肝转移的结肠癌患者的正常组织、结肠癌原发灶及肝转移灶进行免疫组织化学染色，结果显示：超过80%的病例的原发灶和转移灶存在Akt2的高表达。为了进一步验证Akt2对细胞侵袭及转移能力的影响，他们用特异性的Akt2 siRNA载体感染已被荧光素标记的肿瘤细胞，并以空转肿瘤细胞为对照，将细胞注入裸鼠脾脏。4周后，实验组裸鼠均未出现肝脏转移，而对照组则出现明显的多发肝脏转移灶。该研究从不同的水平说明了Akt2在促进结肠癌形成和转移中的重要作用，与其在前列腺癌及脑胶质细胞瘤中的结果基本一致^[12, 13]^。

关于Akt在NSCLC中的研究，有学者发现NSCLC组织中，*Akt2*基因的重要功能位点（激酶区）低突变，而Akt1与Akt3则未见突变，提示*Akt2*基因突变可能是促进肺癌发生的重要原因^[14]^。Nishioka等^[15]^用烟草的主要致癌物质之一亚硝胺吡啶基丁酮（NNK）处理小鼠支气管上皮细胞，1小时后即检测到细胞中的Akt被激活，形成磷酸化Akt（phosphorylated Akt, p-Akt）。Binaifer等^[16]^利用免疫组织化学染色技术检测p-Akt在支气管上皮细胞中的表达，发现16例发育不良或化生的支气管上皮组织中p-Akt为阳性，9例增生组织呈弱阳性，而所有的正常组织则均为阴性。进一步对110例NSCLC组织进行分析，结果显示NSCLC中p-Akt的阳性率为51%（56/110），明显高于正常组织，且免疫组织化学染色提示56例肿瘤组织均表达p-Akt2。以上研究提示：Akt尤其是Akt2的活化可能是导致支气管上皮细胞恶变的重要早期事件。

Park等^[17]^将细胞间粘附分子-3（intercellular adhesion molecule -3, ICAM-3）基因过表达载体导入NSCLC NCI-H1299细胞株，发现细胞中的Akt被激活，继而细胞的迁移和侵袭能力明显增加。我国学者^[18]^也证实肺腺癌细胞的Akt的活化与细胞的侵袭能力呈正相关。另有动物试验^[16]^显示，p-Akt不仅能在体外促进细胞的迁移，而且还能在体内增强气道上皮细胞对粘膜的侵蚀，促进气道肿瘤的生长。

关于Akt与NSCLC患者预后的关系，目前的研究结果尚不完全一致。Shah等^[19]^分析了82例NSCLC患者病灶组织中p-Akt的表达与患者术后生存时间的关系，结果显示：p-Akt高表达组较低表达组生存时间更长，差异有统计学意义（*P*=0.007）；而另一项纳入335例NSCLC患者的研究^[20]^结果则与之相反，单因素和多因素分析均提示：高水平的p-Akt是患者预后不良的独立危险因素。然而，Binaifer^[16]^的研究发现，在110例NSCLC患者中，p-Akt阳性组与阴性组的中位生存时间差异无统计学意义（23个月*vs* 26个月，*P*=0.796 4）。

## 2 Akt上游信号通路蛋白分子——PDK1的研究现状

业已证明，Akt属酯类激酶家族，其活化依赖于上游信号分子的作用。当细胞受到胰岛素样生长因子、血小板源性生长因子等胞外信号刺激时，PI3K的P110催化亚基被激活，磷酸化二磷酸酯酰肌醇[phosphtidylinositol (4, 5)-bisphosphate, PIP2]生成三磷酸酯酰肌醇[phos-phatidylinositol (3, 4, 5)- triphosphate, PIP3]，诱导无活性的Akt和PDK1从细胞质易位至细胞膜，同时使Akt的构象发生改变，暴露出Thr308和Ser473磷酸化位点。位于细胞膜的Akt与PDK1相互靠近，PDK1催化Akt磷酸化，使其活化^[21]^，见图 2。由此可见，PDK1是Akt的重要上游调节分子。

**2:**
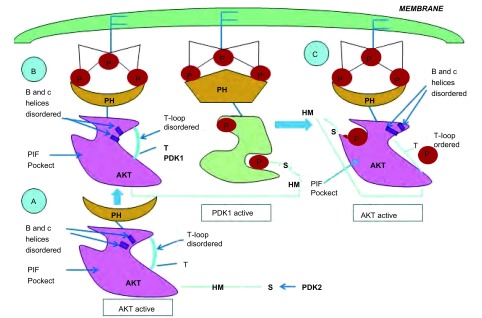
PDK1的作用机制 Mechanism of the action of PDK1

Liu等^[22]^观察到EGF诱导细胞中PDK1和Akt共易位。当用PDK1 siRNA干扰乳腺癌细胞PDK1的表达时，Akt的磷酸化水平明显降低，且细胞的转移和趋化能力明显受损。在裸鼠体内，PDK1 siRNA干扰的乳腺癌细胞成瘤速度缓慢，且不能形成肺部转移病灶。Lee等^[23]^用PDK1抑制剂OSU-03012处理恶性神经鞘瘤细胞，亦发现细胞的增殖减慢，凋亡增加，体内成瘤能力下降。

然而，目前鲜有关于PDK1在NSCLC中的作用及其对Akt的调节机制的报道。笔者所在课题组在前期研究中发现PDK1和Akt在NSCLC组织中的表达均增高，推测PDK1可能参与Akt的活化，进而影响NSCLC的发生。

## 3 Akt下游信号通路蛋白分子——Raf-1和p70S6K的研究现状

现已证实，*Raf*是一种原癌基因，其编码产物为丝氨酸/苏氨酸蛋白激酶，主要参与Ras/Raf/MEK/ERK信号通路的传导。Raf有3种同型异构体，分别为A-Raf、B-Raf和C-Raf（Raf-1）。B-Raf的作用机制目前已较清楚，而Raf-1的功能则了解较少，一般认为与细胞的抗凋亡作用有关^[24]^。近年来，有学者研究发现，Raf-1除了通过Ras/Raf/MEK/ERK通路发挥作用外，还存在非MEK/ERK依赖机制。Raf-1被Akt磷酸化后，在调节细胞增殖、分化和凋亡等方面发挥重要作用^[25]^。p70S6K是Raf-1的作用底物之一，它是核糖体40s小亚基S6蛋白激酶，被Raf-1活化后，促进含5’-末端寡聚嘧啶（5’-terminal oligopolypyrimidine, 5’-TOP）mRNA的翻译，刺激细胞生长、增殖等^[26]^。

Jilavean等^[27]^检测了263例痣和523例恶性黑色素瘤组织中Raf-1的表达，发现肿瘤组织Raf-1的水平较痣明显增高（*P* < 0.000 1），且转移灶较原发灶更高（*P*=0.022 5）。用Raf-1 siRNA干扰恶性黑色素瘤细胞株Raf-1的表达，细胞凋亡增加。Lyustikman等^[28]^的研究结果也显示：神经胶质瘤组织中Raf-1的表达增高；将Raf-1重组逆转录病毒导入小鼠神经胶质细胞，细胞恶变形成神经胶质瘤。目前尚未在NSCLC组织中检测到*Raf-1*的基因突变；*Raf-1*转基因小鼠的肺内腺瘤形成的发生率也较低，存在时间亦较短^[29]^。然而，Cekanov等^[30]^利用烟草致癌原NNK诱导仓鼠发生肺腺癌，并用NNK处理人气道上皮细胞。5周、10周和20周后，仓鼠肺微腺瘤及人气道上皮细胞均被检出有Raf-1的高表达，且呈时间依赖性增加。该实验提示Raf-1可能与肺腺癌的形成有关，是肺腺癌发生的早期标志物之一。实际上，早在20世纪90年代，就有学者利用反义寡核苷酸技术来特异性阻断NSCLC组织中Raf-1的表达，结果发现大部分细胞出现包括染色质浓缩、DNA断裂、annexin Ⅴ结合等在内的凋亡现象，最终细胞死亡^[31]^。Kerkhoff等^[29]^的分析进一步显示：肺癌细胞株中Raf-1蛋白激酶表达水平的升高可能导致细胞恶性转化。因此临床上出现了利用Raf激酶抑制剂BAY 43-9006治疗NSCLC的方法。2005年在美国临床肿瘤学会（American Society of Clinical Oncology, ASCO）会议上，Adjei等^[32]^首次报道了12例NSCLC患者的BAY43-9006 Ⅰ期临床试验结果：1例患者达部分缓解；8例（67%）患者处于疾病稳定期，其中2例长达24周。

p70S6K在乳腺癌及消化道肿瘤中的作用研究较多，而其与NSCLC的关系则报道相对较少。多项研究^[33-35]^表明，p70S6K在乳腺癌、肝癌、食道癌、胆囊癌等肿瘤组织中高表达，且磷酸化水平与肿瘤转移、术后复发或死亡等明显正相关。p70S6K对NSCLC意义的研究中，Shen等^[36]^用高通量免疫印迹、Western blot和免疫组织化学染色等方法检测肺腺癌和正常支气管上皮组织中p70S6K的表达水平，发现p70S6K在肺腺癌组织中增高，故其在肺腺癌的鉴别诊断中有一定价值。本课题组在前期研究中探讨了Raf-1和p70S6K在不同病理类型NSCLC中的表达，结果显示Raf-1在肺腺癌和鳞癌组织中均呈高表达；而p70S6K则在肺腺癌组织中表达增高，在鳞癌组织中阴性表达。因此，Akt/ Raf-1在肺腺癌和鳞癌组织中是否均通过作用于底物p70S6K而发挥作用尚需进一步的研究和分析。

## 4 小结

综上所述，现有的研究已经提示PDK1、Akt、Raf-1和p70S6K四种蛋白质参与了包括NSCLC在内的多种肿瘤的发生。然而，以Akt为中心的PDK1/Akt/p70S6K信号通路在NSCLC中的作用机制尚有待于系统的深入研究，以期进一步明确NSCLC的发生机制，探寻NSCLC早期诊断的分子标志物和靶向治疗新靶点。
